# Supplementary material for: Stimulated plasmon polariton scattering
Source: Nat Commun. 2020 Aug 12;11:4039. doi: 10.1038/s41467-020-17810-4 (PMC7423978; doi:10.1038/s41467-020-17810-4)
Supplement: Supplementary file 1 — Supplementary Information [file 41467_2020_17810_MOESM1_ESM.pdf]

SUPPLEMENTARY NOTE 1: THEORY

A. Introduction & Preliminaries

In this supplemantary note, we present the theoretical foundation for the paper "Stimulated Plasmon Polariton Scattering". This is mainly a derivation of the coupled mode equations Eq. (1a-c) of the main paper and the expressions for the nonlinear coupling and total gain. This part follows in broad strokes an earlier paper of ours on the theory of Brillouin scattering [1]. Furthermore, we discuss important aspects such as the normalization of the polaritonic modes.

We consider an optical waveguide along the y-direction of a Cartesian system. It is composed of high-index materials as described by the permittivity $\varepsilon(x, z)$ in the transverse plane and optionally a Pockels-nonlinearity $\chi^{(2)}(x, z)$. We assume that the waveguide supports bound propagating modes. Furthermore, we assume that waveguide modes interact with a nearby material that supports low-frequency plasmon-polariton modes. In the following we will assume this to be graphene, but other materials from the wider class of van-der-Waals materials or systems such as a 2d electron gas confined in a quantum well structure are also promising candidates, but might require minor modifications to the formalism. We assume that the plasma frequency in the polaritonic material can be tuned. In the case of graphene this is via the Fermi energy E_F, e. g. through adsorption of surface dopants or via an external gate electrode.

In the following, we will use upper case letters $\mathbf{E}$ and $\mathbf{H}$ for physically observable (real-valued) electromagnetic fields. Corresponding modal quantities $\mathbf{e}$, $\mathbf{h}$ are written in lower case. In order to simplify notation, we employ the Einstein summation convention where advantageous.

1. *Optical modes*

Inside the waveguide, we assume a forward-propagating pump wave with angular frequency ω and wave number k as well as a slightly red-shifted counter-propagating Stokes wave. We assume that the propagation loss of the optical modes is weak enough to be captured via perturbation theory within a coupled mode framework based on the solutions to the lossless optical wave equations

$$\nabla \times \nabla \times \mathbf{E} = -\mu_0 \partial_t^2 \mathbf{D}; \tag{1}$$

where $\mathbf{E}$ and $\mathbf{D} = \varepsilon\mathbf{E}$ are the electric field and the electric displacement, $\varepsilon(\mathbf{r})$ is the permittivity distribution and all materials are assumed to have the vacuum permeability μ_0. As basis functions for the subsequent coupled mode theory, we neglect the imaginary part of ε and solve for stationary modes

$$\mathbf{E}^{(p)}(\mathbf{r}, t) \approx a^{(p)}(y)\mathbf{e}^{(p)}(x, z) \exp(\mathrm{i}ky - \mathrm{i}\omega t) + \text{c.c.}, \tag{2}$$

$$\mathbf{E}^{(s)}(\mathbf{r}, t) \approx a^{(s)}(y)\mathbf{e}^{(s)}(x, z) \exp(-\mathrm{i}ky - \mathrm{i}\omega t) + \text{c.c.}, \tag{3}$$

where $\mathbf{e}^{(p)}$ and $\mathbf{e}^{(s)}$ are the modal profiles of the forward-propagating pump and backward-propagating Stokes wave, respectively, and $a^{(p)}$ and $a^{(s)}$ are the corresponding mode amplitudes. For any but the simplest geometries the mode distributions $\mathbf{e}^{(p/s)}(x, z)$ must be calculated numerically e. g. using finite elements.

Since we are investigating a nonlinear process, the relevant equations are not independent of amplitude levels. Therefore, the optical modes must be normalized, e. g. with respect to the energy density

$$\mathcal{E}_a = 2 \int \mathrm{d}^2 r\ \varepsilon [\mathbf{e}^{(p)}]^* \cdot \mathbf{e}^{(p)} \tag{4}$$

$$= 2 \int \mathrm{d}^2 r\ \varepsilon [\mathbf{e}^{(s)}]^* \cdot \mathbf{e}^{(s)}, \tag{5}$$

where $\mathcal{E}_a$ is the unit of energy per unit length of waveguide, whose value is in principle arbitrary; typical choices are based on energies of one Joule or one optical quantum $\hbar\omega$. The energy density is connected to the total power flux $\mathcal{P}_a$ via the group velocity v_a:

$$\mathcal{P}_a = v_a \mathcal{E}_a. \tag{6}$$

2. *Polaritonic modes*

In analogy to the optical modes, we now describe the plasmon polaritons inside the graphene sheet in terms of modes based on similar assumptions as for the optical waveguide. We assume that the sheet supports a tightly localized polariton mode at angular frequency Ω and wave number q. In the regime of strong confinement, the field distribution of the polariton of a sheet that is situated at an interface between two dielectrics with permittivities ε_1 and ε_2 can be approximated as:

$$\mathbf{E}^{(\mathrm{pol})}(\mathbf{r}, t) \approx b(y)\mathbf{e}^{(\mathrm{pol})}(x, z) \exp(\mathrm{i}qz - \mathrm{i}\Omega t) + \text{c.c.}, \tag{7}$$

$$\mathbf{e}^{(\mathrm{pol})}(x, z) \approx \begin{pmatrix} 0 \\ \pm 1 \\ \mathrm{i} \end{pmatrix} E_0 \exp[-q|z|)], \tag{8}$$

where E_0 is the longitudinal electric field amplitude at the sheet. The plus and minus signs apply above and below the sheet, respectively; the total discontinuity of the normal electric field component is given as $\Delta E = 2E_0$.

The modal energy per unit length and unit width of the sheet material (required for normalization of the coupled mode equations) comprises three parts: the electromagnetic energy in the dielectrics surrounding the sheet, the electromagnetic energy inside the sheet (which is of similar order) and the non-electromagnetic energy of the electron system of the sheet:

$$\mathcal{E}_b = \underbrace{\frac{2\varepsilon_0(\varepsilon_1 + \varepsilon_2)E_0^2}{q}}_{\text{outside sheet}} + \underbrace{\frac{\Im\{\sigma(\Omega)\}E_0^2}{\Omega}}_{\text{inside sheet}} + \mathcal{E}_{\mathrm{int}}, \tag{9}$$

While the first two terms are universal in as far as they only require knowledge of the polaritonic dispersion relation [which is equivalent to the knowledge of $\sigma(\Omega)$], the third term is highly specific for the particular choice of material. For illustration purposes, we calculate the non-electromagnetic correction for a graphene plasmon polariton due to the degeneracy pressure of the free carriers at zero temperature in the next section. At room temperature, we expect the main findings to remain qualitatively valid, but stress that the electromagnetic parts must be recalculated with the finite-temperature conductivity.

3. *Graphene*

Ignoring nonlocal effects inside the graphene and assuming zero temperature for simplicity, its electromagnetic properties are characterized by a dispersive sheet conductance:

$$\sigma(\Omega) = \sigma_1(\Omega) + \sigma_2(\Omega), \tag{10}$$

$$\sigma_1(\Omega) = \frac{\mathrm{i}e^2 E_F}{\pi\hbar^2(\Omega + \mathrm{i}\gamma)}, \tag{11}$$

$$\sigma_2(\Omega) = \frac{e^2}{4\hbar^2}\left[\Theta(\hbar\Omega - 2E_F) + \frac{\mathrm{i}}{\pi}\ln\left|\frac{\hbar\Omega - 2E_F}{\hbar\Omega + 2E_F}\right|\right], \tag{12}$$

where e is he the electron charge, $\hbar$ is the Planck constant and Θ is the Heaviside function. Here, σ_1 describes the contribution to the overall conductivity from intra-band effects of the free carriers, whereas σ_2 describes inter-band transitions inside the graphene sheet.

We now calculate the correction of the total polaritonic energy due to the degeneracy pressure of the electron gas. To this end, we assume that the carrier density is a harmonic variation around an equilibrium value defined by the mean Fermi energy:

$$n(t) = n_0[1 + \alpha\sin(\Omega r)], \tag{13}$$

where α is a small parameter. The 2d energy density associated with a carrier density is given via the chemical potential $\mu(n) = \hbar v_{\mathrm{F}}\sqrt{\pi n}$:

$$\mathcal{E}_{\mathrm{deg}}(t) = \int_0^{n(t)} \mathrm{d}n'\ \mu(n') = \frac{2\hbar v_{\mathrm{F}}\sqrt{\pi n(t)^3}}{3}. \tag{14}$$

The degeneracy correction to the modal energy is the average of this expression over one polaritonic cycle:

$$\mathcal{E}_{\mathrm{deg}} = \frac{\Omega}{2\pi}\int_0^{2\pi/\Omega} \mathrm{d}t\ \mathcal{E}_{\mathrm{deg}}(t) \tag{15}$$

$$= \frac{2\hbar v_{\mathrm{F}}\sqrt{\pi n_0^3}}{3}\left[1 + \frac{3\alpha^2}{16} + \mathcal{O}(\alpha^4)\right]. \tag{16}$$

$$= \mathcal{E}_{\mathrm{deg}}^{(0)} + \mathcal{E}_{\mathrm{int}} + \mathcal{O}(\alpha^4). \tag{17}$$

Here, $\mathcal{E}_{\mathrm{deg}}^{(0)}$ is the energy introduced by doping, whereas the leading order correction with respect to α is taken as the modal energy of the polariton. By relating the variation αn_0 of the charge carrier density to the discontinuity of the electric normal field via Maxwell's divergence equation

$$\varepsilon_0(\varepsilon_1 + \varepsilon_2)E_0 = \alpha e n_0, \tag{18}$$

we can express the degeneracy correction in terms of the polaritonic field amplitude:

$$\mathcal{E}_{\mathrm{int}} = \frac{\pi[\hbar v_{\mathrm{F}}\varepsilon_0(\varepsilon_1 + \varepsilon_2)]^2}{8e^2 E_F}E_0^2. \tag{19}$$

We find for the total polaritonic energy per unit area of graphene:

$$\mathcal{E}_b = \Bigg[\overbrace{\frac{4\varepsilon_0(\varepsilon_1 + \varepsilon_2)}{q}}^{\text{e.g. } 2.0\times10^{-16}\,\mathrm{F}} + \overbrace{\frac{2\Im\{\sigma(\Omega)\}}{\Omega}}^{\text{e.g. } 1.0\times10^{-16}\,\mathrm{F}} + \underbrace{\frac{2\pi[\hbar v_{\mathrm{F}}\varepsilon_0(\varepsilon_1 + \varepsilon_2)]^2}{8e^2 E_F}}_{\text{e.g. } 2.8\times10^{-19}\,\mathrm{F}}\Bigg]\frac{E_0^2}{2}, \tag{20}$$

where the prefactor takes on the meaning of an effective capacitance. Numerical values for the respective contributions have been evaluated for for the example $\varepsilon_1 = 1$, $\varepsilon_2 = 12$, $E_F = 0.1\,\mathrm{eV}$, $\Omega = 0.1E_F/\hbar = 1.5\times10^{13}\mathrm{s}^{-1}$, $q = 2.3\times10^6\,\mathrm{m}^{-1}$. Clearly, this formula is reliable only in a low-loss regime. However, we can see that the in-sheet correction to the electromagnetic energy cannot be neglected, whereas the correction due to degeneracy pressure is (at least for this example) minimal.

B. Modal equations

We now derive nonlinear coupled mode equations for the stimulated plasmon polariton scattering problem.

1. *Basic coupled mode equations*

The optical equation of motion can be obtained from the wave equation:

$$\nabla\times\nabla\times\mathbf{E} = -\mu_0\partial_t^2\mathbf{D}. \tag{21}$$

To this end, we first assume that the electric field is the sum of the two optical modes

$$\mathbf{E} = a^{(\mathrm{p})}(y)\mathbf{e}^{(\mathrm{p})}\exp(\mathrm{i}ky - \mathrm{i}\omega t) + a^{(\mathrm{s})}(y)\mathbf{e}^{(\mathrm{s})}\exp(-\mathrm{i}ky - \mathrm{i}\omega t) + \mathrm{c.c.}. \tag{22}$$

In contrast, the electric displacement is the sum of the optical modes and a nonlinear polarization $\mathbf{P}^{(\mathrm{NL})}$:

$$\mathbf{D} = \varepsilon a^{(\mathrm{p})}(y)\mathbf{e}^{(\mathrm{p})}\exp(\mathrm{i}ky - \mathrm{i}\omega t) + \varepsilon a^{(\mathrm{s})}(y)\mathbf{e}^{(\mathrm{s})}\exp(-\mathrm{i}ky - \mathrm{i}\omega t) + \mathrm{c.c.} + \mathbf{P}^{(\mathrm{NL})}. \tag{23}$$

We then insert this ansatz into the wave equation and project back onto either optical mode $\mathbf{e}^{(\mathrm{p/s})}$. We average over a time scale much larger than an optical cycle in order to isolate the dynamics of the slowly varying envelopes $a^{(\mathrm{p/s})}$ and we neglect second-order derivatives. We thus arrive at the optical equations of motion:

$$\partial_y a^{(\mathrm{p})} + v_a^{-1}\partial_t a^{(\mathrm{p})} + \kappa_a a^{(\mathrm{p})} = -\mathrm{i}\omega \mathcal{P}_a^{-1}\langle \mathbf{e}^{(\mathrm{p})}|\mathbf{P}^{(\mathrm{NL})}\rangle \tag{24}$$

$$\partial_y a^{(\mathrm{s})} - v_a^{-1}\partial_t a^{(\mathrm{s})} - \kappa_a a^{(\mathrm{s})} = -\mathrm{i}\omega \mathcal{P}_a^{-1}\langle \mathbf{e}^{(\mathrm{s})}|\mathbf{P}^{(\mathrm{NL})}\rangle \tag{25}$$

Here, $\mathcal{P}_a$ appears from the overlap integral of the optical mode with itself and the source terms are the phase-matched projections of the nonlinear polarization onto the optical modes. The decay parameter κ_a is given as the overlap between the optical mode and the dielectric loss of the waveguide:

$$\kappa_a = \int \mathrm{d}^2 r\ \mathbf{e}^{*(\mathrm{p})}\Im\{\varepsilon\}\mathbf{e}^{(\mathrm{p})}. \tag{26}$$

In essentially the same way, the equation of motion is derived for the plasmon polariton envelope $b(y)$ from the optical wave equation in the THz range:

$$\begin{aligned}\partial_y b + v_b^{-1}\partial_t b + \kappa_b b = &- \mathrm{i}\Omega Q^* \mathcal{P}_b^{-1}\big[\langle \mathbf{e}^{(\mathrm{pol})}|\mathbf{S}^{(E)}\rangle \\ &+ \langle n^{(\mathrm{pol})}|S^{(n)}\rangle\big],\end{aligned} \tag{27}$$

where κ_b describes the absorption of the polaritonic mode along the sheet due to the real part of the sheet conductance $\sigma(\Omega)$ and $\mathbf{e}^{(\mathrm{pol})}$ and $n^{(\mathrm{pol})}$ are the electric field distribution and carrier density distribution of the polaritonic mode. The source terms $\mathbf{S}(\mathbf{r},t)^{(E)}$ and $S(\mathbf{r},t)^{(n)}$ to the polaritonic mode due to the optical pumping will be discussed in the next section.

The perturbative treatment of the losses is of course not as good an approximation for the polaritonic amplitude as it is for the optical ones, but should suffice for our purposes. This is based on the experience with the problem stimulated Brillouin scattering, where the lossless mode approximation is very successfully applied to the acoustic part of the problem, which features typical quality factors of several 100 to several 1000 just as we predict in the present work.

2. *Nonlinear coupling*

We assume that the coupling between the optical and polaritonic modes is due to three-wave mixing via some second order nonlinearity somewhere in the system. Therefore, we assume that the nonlinear polarization is the mixing product between the total optical field and the polaritonic field:

$$P_l{}^{(\mathrm{NL})} = \big(\mathbf{E}_m{}^{(\mathrm{p})} + \mathbf{E}_m{}^{(\mathrm{s})}\big)\big(\varepsilon_0\chi^{(2)}_{lmn}\mathbf{E}_n{}^{(\mathrm{pol})} + \Pi_{lm}n^{(\mathrm{pol})}\big), \tag{28}$$

where $\chi^{(2)}$ is the conventional second-order susceptibility e. g. due to the Pockels effect in the waveguide material and $n(\mathbf{r})$ is the local deviation of the carrier density from the average in the graphene sheet. The symbol Π is describes the ponderomotive effect in graphene and only introduced here to keep he notation manageable and highlight the structure of the equations; in the next section we will not use it further and instead derive the ponderomotive expressions explicitly. Analogously, we assume that the polaritonic driving term is the mixing product between the two slightly detuned optical fields:

$$S_l^{(E)} = \chi^{(2)}_{lmn}\mathbf{E}_m{}^{(\mathrm{p})}\mathbf{E}_n{}^{(\mathrm{s})}, \tag{29}$$

$$S^{(n)} = \Pi_{mn}\mathbf{E}_m{}^{(\mathrm{p})}\mathbf{E}_n{}^{(\mathrm{s})}. \tag{30}$$

When evaluating the overlap products on the right hand sides of the coupled mode equations and performing the time averages, we find:

$$\begin{aligned}\langle \mathbf{e}^{(\mathrm{p})}|\mathbf{P}^{(\mathrm{NL})}\rangle = a^{(\mathrm{s})}b\int \mathrm{d}^2 r\ \big[&\chi^{(2)}_{lnm}e_l^{*(\mathrm{p})}e_m{}^{(\mathrm{s})}e_n{}^{(\mathrm{pol})} \\ &+ \Pi_{lm}e_l^{*(\mathrm{p})}e_m{}^{(\mathrm{s})}n^{(\mathrm{pol})}\big],\end{aligned} \tag{31}$$

$$\begin{aligned}\langle \mathbf{e}^{(\mathrm{s})}|\mathbf{P}^{(\mathrm{NL})}\rangle = a^{(\mathrm{p})}b^*\int \mathrm{d}^2 r\ \big[&\chi^{(2)}_{lnm}e_l^{*(\mathrm{s})}e_m{}^{(\mathrm{p})}e_n^{*\,(\mathrm{pol})} \\ &+ \Pi_{lm}e_l^{*(\mathrm{s})}e_m{}^{(\mathrm{p})}n^{*(\mathrm{pol})}\big],\end{aligned} \tag{32}$$

$$\langle \mathbf{e}^{(\mathrm{pol})}|\mathbf{S}\rangle = a^{(\mathrm{p})}a^{*(\mathrm{s})}\int \mathrm{d}^2 r\ \chi^{(2)}_{lnm}e_l^{*(\mathrm{pol})}e_m^{*\,(\mathrm{s})}e_n{}^{(\mathrm{p})} \tag{33}$$

$$\langle n^{(\mathrm{pol})}|\mathbf{S}\rangle = a^{(\mathrm{p})}a^{*(\mathrm{s})}\int \mathrm{d}^2 r\ \Pi_{lm}n^{*(\mathrm{pol})}e_l^{*(\mathrm{s})}e_m{}^{(\mathrm{p})}. \tag{34}$$

By introducing the coupling integral

$$\begin{aligned}Q = \int \mathrm{d}^2 r\ \big[&\chi^{(2)}_{lnm}e_l^{*(\mathrm{p})}e_m{}^{(\mathrm{s})}e_n{}^{(\mathrm{pol})} \\ &+ \Pi_{lm}e_l^{*(\mathrm{p})}e_m{}^{(\mathrm{s})}n^{(\mathrm{pol})}\big],\end{aligned} \tag{35}$$

we obtain the final coupled mode equations:

$$\partial_y a^{(\mathrm{p})} + v_a^{-1}\partial_t a^{(\mathrm{p})} + \kappa_a a^{(\mathrm{p})} = -\mathrm{i}\omega Q \mathcal{P}_a^{-1}a^{(\mathrm{s})}b^*, \tag{36}$$

$$\partial_y a^{(\mathrm{s})} - v_a^{-1}\partial_t a^{(\mathrm{s})} - \kappa_a a^{(\mathrm{s})} = -\mathrm{i}\omega Q^* \mathcal{P}_a^{-1}a^{(\mathrm{p})}b, \tag{37}$$

$$\partial_y b + v_b^{-1}\partial_t b + \kappa_b b = -\mathrm{i}\Omega Q \mathcal{P}_b^{-1}[a^{(\mathrm{p})}]^* a^{(\mathrm{s})}, \tag{38}$$

The most natural source for a second order nonlinearity in the system is of course the Pockels effect. This can be introduced, e. g. by composing the waveguide at least partially of a nonlinear material such as lithium niobate. We cannot say much more about such a setup except that we could not obtain an appreciable SPPS-gain based on this effect alone. The problem is that the polaritonic mode does not penetrate the waveguide enough to lead to a sufficient nonlinear mode overlap. We do, however, emphasize that this approach might prove very useful for the excitation of polaritons in materials other than graphene.

At least in the case of graphene the dominant second-order nonlinearity for the SPPS-process turns out to be the ponderomotive interaction, i. e. the local shift of the Fermi energy inside the graphene sheet as a result of the beat between the optical fields and – conversely – the emergence of a dynamic grating in the graphene conductance from a plasmon polariton.

3. Ponderomotive nonlinearity in graphene

We consider the case of a polariton in the THz-range and ask how this modulates the optical conductivity of graphene. This is a second-order nonlinearity, but not a conventional Pockels nonlinearity. The reason is that the polaritonic mode has even symmetry with respect to the sheet plane while the Pockels effect would describe the impact of a homogeneous electric field (which has odd symmetry) on the conductivity. Therefore (and unlike a Pockels nonlinearity) this particular type of second-order nonlinearity is not symmetry-forbidden in a system with inversion symmetry. One characteristic of materials like graphene is the very strong optical anisotropy; while the electrons are nearly free within the plane of the sheet, they can barely move in the normal direction. As a result, the ponderomotive effect arising from the plasma properties of the electron system interacts only with the in-plane components of the electric field.

The idea is that the polaritonic mode is in essence a fluctuation in carrier density, and therefore accompanied by a spatial modulation of the Fermi level E_F. This clearly implies the assumption that the polariton does not cause too much unrest in the electron system, but rather moves carriers around in a quasi-adiabatic way. The Fermi level in turn controls the intra-band conductance of graphene through

$$\sigma_1(\omega) = \frac{\mathrm{i}e^2}{\pi\hbar^2(\omega + \mathrm{i}\gamma)} \cdot E_F, \tag{39}$$

where ω is the optical angular frequency. The Fermi-level is also linked to the total carrier density n_{tot}:

$$E_F = \hbar v_{\mathrm{F}}\sqrt{\pi n_{\mathrm{tot}}} = \hbar v_{\mathrm{F}}\sqrt{\pi(n_0 + n)}, \tag{40}$$

where n_0 is the equilibrium carrier density due to doping and n is the carrier density due to the polariton. The latter finally depends on the electric field discontinuity across the sheet:

$$n = \frac{q\varepsilon_0(\varepsilon_1 + \varepsilon_2)E_0}{e}, \tag{41}$$

where q is the wave number of the polariton and doubles as the localization length of the electric field normal to the sheet. The nonlinearity in question (evaluated at $n = 0$) is then:

$$\frac{\partial\sigma_1}{\partial E_0} = \underbrace{\frac{\mathrm{i}e^2}{\pi\hbar^2(\omega + \mathrm{i}\gamma)}}_{(\partial\sigma_1)/(\partial E_F)} \cdot \underbrace{\frac{\hbar v_{\mathrm{F}}\sqrt{\pi}}{2\sqrt{n_0}}}_{(\partial E_F)/(\partial n)} \cdot \underbrace{\frac{q\varepsilon_0(\varepsilon_1 + \varepsilon_2)}{e}}_{(\partial n)/(\partial E_0)}. \tag{42}$$

With the Fermi wave number $q_F = \sqrt{\pi n_0}$, this can be simplified:

$$\frac{\partial\sigma_1}{\partial E_0} = \frac{e\varepsilon_0 v_{\mathrm{F}}}{2\hbar} \cdot \frac{\mathrm{i}(\varepsilon_1 + \varepsilon_2)}{\omega + \mathrm{i}\gamma} \cdot \frac{q}{q_F}. \tag{43}$$

It should be repeated that q and ω characterize different waves: q is the polaritonic wave number and ω is the optical angular frequency. In summary, we have found that a plasmon polariton in a graphene sheet is accompanied by an optical grating with the same period as the plasmon polariton. This dynamic grating can back-scattering light from the optical pump mode into the optical Stokes mode and thereby mediate stimulated plasmon polariton scattering.

The complementary effect (the source term to the polaritonic excitation due to the optical pumps) is derived from the same mathematical quantity $(\partial\sigma)/(\partial E_0)$, but with a different physical interpretation as a ponderomotive force in the 2d electron plasma. At this point we forgo an in-depth discussion of the connection between dynamic grating effect and the ponderomotive force via Onsager relations as well as the derivation of the inter-band contributions to this effect at finite temperature. Instead, we refer to our recent work [2] and only state the expressions that are relevant for the present work. The full optical conductivity of graphene at finite temperature has been calculated in Refs. [3, 4]

$$\begin{aligned}\frac{\sigma^{(T>0)}(\omega)}{\sigma_K} =& \frac{2\mathrm{i}E_F}{\hbar(\omega + \mathrm{i}\gamma)} + \frac{\pi}{4}\Bigg(\tanh\frac{\hbar\omega + 2E_F}{4k_{\mathrm{B}}T} \\ &+ \tanh\frac{\hbar\omega - 2E_F}{4k_{\mathrm{B}}T} \\ &+ \frac{\mathrm{i}}{\pi}\ln\frac{(\hbar\omega - 2E_F)^2 + (2k_{\mathrm{B}}T)^2}{(\hbar\omega + 2E_F)^2}\Bigg),\end{aligned} \tag{44}$$

where $\sigma_K = e^2/h \simeq 3.87 \times 10^{-5}\,\mathrm{S}$ is the inverse von-Klitzing constant, k_{B} is the Boltzmann constant. From this, we derived the ponderomotive nonlinearity

$$\begin{aligned}\frac{\partial\sigma^{(T>0)}}{\partial E_0} =& \frac{1}{2} \cdot \frac{\partial\sigma^{(1)}}{\partial E_0} \cdot \Bigg[2 + \frac{\hbar\omega}{\hbar\omega + 2E_F} \\ &+ \frac{\hbar\omega(\hbar\omega - 2E_F)}{(\hbar\omega - 2E_F)^2 + (2k_{\mathrm{B}}T)^2}\Bigg],\end{aligned} \tag{45}$$

expressed in terms of the quantity given in Eq. (43) for simplicity. In this form, the square brackets express the fact that the inter-band threshold leads to a resonant enhancement of the ponderomotive force, which translates to a peak in the SPPS response as reported in the main text.

Power gain and gain scaling

We now derive the expression presented in the main paper for the total SPPS-gain. To this end, we start with the coupled mode equations

$$\partial_y a^{(\mathrm{p})} + v_a^{-1}\partial_t a^{(\mathrm{p})} + \kappa_a a^{(\mathrm{p})} = -\mathrm{i}\omega Q\mathcal{P}_a^{-1}a^{(\mathrm{s})}b^*, \tag{46}$$

$$\partial_y a^{(\mathrm{s})} - v_a^{-1}\partial_t a^{(\mathrm{s})} - \kappa_a a^{(\mathrm{s})} = -\mathrm{i}\omega Q^*\mathcal{P}_a^{-1}a^{(\mathrm{p})}b, \tag{47}$$

$$\partial_y b + v_b^{-1}\partial_t b + \kappa_b b = -\mathrm{i}\Omega Q\mathcal{P}_b^{-1}[a^{(\mathrm{p})}]^*a^{(\mathrm{s})}, \tag{48}$$

and assume both steady state and a local polaritonic response. The latter is justified if the plasmon polariton propagation length is smaller than the expected length scale on which the optical amplitudes change. This is justified in the case presented in the main paper. Due to the steady state assumption, all time derivatives vanish and the local polaritonic response assumption also removes the spatial derivative in the equations for $b(y)$. Therefore the polaritonic amplitude can be explicitly solved:

$$b = -\mathrm{i}\frac{\Omega Q}{\mathcal{P}_b \kappa_b}[a^{(\mathrm{p})}]^* a^{(\mathrm{s})}. \tag{49}$$

By inserting this in the steady-state optical equations, we find:

$$\partial_y a^{(\mathrm{p})} + \kappa_a a^{(\mathrm{p})} = \frac{\omega\Omega|Q|^2}{\mathcal{P}_a \mathcal{P}_b \kappa_b} \left|a^{(\mathrm{s})}\right|^2 a^{(\mathrm{p})}, \tag{50}$$

$$\partial_y a^{(\mathrm{s})} - \kappa_a a^{(\mathrm{s})} = -\underbrace{\frac{\omega\Omega|Q|^2}{\mathcal{P}_a \mathcal{P}_b \kappa_b}}_{=\Gamma} \left|a^{(\mathrm{p})}\right|^2 a^{(\mathrm{s})}, \tag{51}$$

where Γ is the amplitude gain coefficient of the SPPS process and implicitly depends on the power unit $\mathcal{P}_a$ used for normalizing the optical modes. In practice, most experiments measure the power gain, which relates optical power levels rather than arbitrarily normalized amplitudes. The power carried by the pump and Stokes modes are given by:

$$P^{(\mathrm{p/s})} = \mathcal{P}_a |a^{(\mathrm{p/s})}|^2. \tag{52}$$

Its y-derivative can be found via the product rule:

$$\partial_y P^{(\mathrm{p/s})} = \mathcal{P}_a a^{*(\mathrm{p/s})} \partial_y a^{(\mathrm{p/s})} + \text{c.c.}. \tag{53}$$

By inserting this in Eqs. (50, 50), we find:

$$\partial_y P^{(\mathrm{p})} + \kappa_a P^{(\mathrm{p})} = \frac{2\omega\Omega|Q|^2}{\mathcal{P}_a^2 \mathcal{P}_b \kappa_b} P^{(\mathrm{s})} P^{(\mathrm{p})}, \tag{54}$$

$$\partial_y P^{(\mathrm{s})} - \kappa_a P^{(\mathrm{s})} = -\underbrace{\frac{2\omega\Omega|Q|^2}{\mathcal{P}_a^2 \mathcal{P}_b \kappa_b}}_{=G} P^{(\mathrm{p})} P^{(\mathrm{s})}, \tag{55}$$

where G finally is the SPPS power gain coefficient as presented in the main paper.

Comparison with SBS

Confusion may arise as to the impact of polaritonic loss in SPPS. One first source of confusion can be the fact that the SPPS amplification effect stretches over a length orders of magnitude greater than the polariton propagation length. The short answer is that while the polariton may only propagate for 1-10 microns, the optical Stokes wave carries its information and together with the pump generates an amplified copy of the polaritonic signal further towards the pump. A second source of confusion can be the general perception that (especially plasmon) polaritons are very lossy while sound is nearly lossless and therefore SBS might seem massively more viable than SPPS. Here, the short answer is that acoustic loss increases dramatically with frequency and SBS involves GHz sound waves with a propagation length of 1-100 microns (depending on the material). We will now provide some discussion of how SBS and SPPS compare.

4. *Comparison of loss figures*

The appropriate measure for the loss of the non-optical field in both SBS and SPPS is the propagation length l. It can be easily found from the quality factor $\mathcal{Q}$ and the acoustic/plasmonic wavenumber q:

$$l = \mathcal{Q}/q.$$

The quality factor in turn can be found either as the ratio of wavenumber and decay constant (just the inverse of the equation above) or the ratio of acoustic frequency and damping rate. From the phase matching diagram Fig.1a of the main text, which is identical for both SBS and SPPS, it is clear that $q \approx 2k$ and therefore very similar in both processes. This reduces the question of propagation lengths to comparing the quality factors Q of both processes. We find Q between 10 and 1000 in SPPS (see Fig.2b). As for the losses in SBS, Boyd [5] lists Stokes frequencies (denoted Ω_B) and damping rates (denoted Γ_B) in various bulk solids and liquids. By dividing Ω_B and Γ_B, one easily verifies that SBS has Q-factors in the same ballpark as SPPS. This also holds for material systems that have been the focus of recent SBS-research such as integrated soft glass waveguides ($\Omega/2\pi = 7.7\,\mathrm{GHz}$, $\Gamma/2\pi = 34\,\mathrm{MHz}$ [6], corresponding to $\mathcal{Q} = 226$) or silicon waveguides (e.g. $\mathcal{Q} = 306$ in Ref. [7]). We find that SBS and SPPS suffer from comparable amounts of loss in the non-optical fields.

C. Competition between loss and nonlinear coupling

The viability of SPPS cannot be shown by comparing its typical linear loss figures with those of SBS. In addition, it is necessary to demonstrate that the nonlinear coupling is also strong enough to be comparable with SBS. The relevant parameter for such a comparison is the gain coefficient G. It actually already includes the loss in the form of the decay constant κ_B [see Eq. (55)]. As a result, G scales with the quality factor and therefore expresses precisely how strong the nonlinearity is compared to the loss of the non-optical wave (sound in SBS, plasmons in SPPS).

Therefore the question how the competition between nonlinear coupling and loss in SPPS compares to that in SBS can be decided by comparing the gain coefficients G. In SBS the gain is stated in one of two ways:

As a material parameter (units m/W) appropriate for quasi-bulk systems such as fibres or as a waveguide parameter (units $(\mathrm{Wm})^{-1}$) appropriate for complex nanoscale systems such as our example. The two gain figures are related by the effective mode area of the optical modes. Boyd lists the former quantity and based on an effective mode area of $0.1, \mu\mathrm{m}^2$ (comparable to the mode area in our example), this translates to gains between $15\,(\mathrm{Wm})^{-1}$ for CS_2 and $\sim 4.5\,\mathrm{Wm}^{-1}$ for water and silica. A further discussion of this can be found in the main manuscript.

SUPPLEMENTARY NOTE 2: POWER HANDLING AND ELECTRON GAS HEATING IN GRAPHENE

Most conventional nonlinear experiments use very large intensities and observe strong heating of the electron gas. For the most part, it is an ultrafast transient effect upon absorbing a tightly focused femtosecond pulse. In contrast, we envisage pulses that are several orders of magnitude longer and that are dissipated along the entire waveguide, which further reduces the local heating effect. This requires a closer look to which extent electron gas heating might be detrimental to our proposal.

The dissipation in the sheet is due to inelastic scattering events between charge carriers and some other (quasi-)particles. Apart from static scattering sources such as impurities, there are mainly two candidates available as scattering partners: phonons and electrons. First, we note that 2d electron systems such as graphene are similar to 3d systems in that within the framework of Fermi liquid theory electron-electron interactions are absorbed in renormalized parameters for the Fermi quasi-particles, which constitute the non-interacting charge carriers of the system. As a result, inelastic carrier-carrier scattering is a very weak process to begin with. Furthermore, the effect of temperature on the carrier population is fundamentally different to the effect on the phonon population due to the Fermionic nature of the former whereas the latter are collective Bosonic excitations with chemical potential zero.

A change in temperature leads to a widening of the carrier distribution with an additional shift in the chemical potential (Fermi level) while maintaining the overall number of particles. As a result, the number of available scattering partners does not change with temperature although the number of available destination states does increase. In contrast, the number of phonons increases with temperature to first approximation according to the Stefan–Boltzmann law (with the third power of temperature in 3d systems), in addition to the aforementioned increase in the number of available destination states. As a result, it is implausible that a high electron gas temperature leads to significantly increased dissipation for electromagnetic excitations in the intra-band regime. In summary, we conclude that electron gas heating an no significant effect on the quality factors of plasmon polaritons in the intra-band regime, which we find for $E_F > 0.1\,\mathrm{eV}$. In this regime the plasmon polariton loss is determined by the temperature of the lattice.

After having established that an elevated electron temperature does not lead to significantly increased Ohmic loss for the plasmon polaritons, we now address what kind of effects it will have, i.e. effects that are directly linked to the softening of the Fermi distribution rather than scattering events. Probably the main effect of the electron temperature is the sharpness of the onset of the inter-band contribution to the conductivity. This manifests in two ways. On the one hand, it leads to a softer transition between the regimes of low and high optical loss. On the other hand, it impacts the maximally attainable gain figure, because the increased gain at the inter-band threshold and at lower temperatures is to large parts due to a resonant ponderomotive nonlinearity at the inter-band threshold. This means that while the electron gas temperature does not influence the quality factor, it does affect the nonlinear coupling between the optical and plasmonic modes as well as the optical propagation length of the hybrid waveguide.

Given that the electron temperature will have an effect on our results, it is necessary to estimate a realistic temperature in settings such as the examples studied in our manuscript. It appears to us that the topic of hot electrons in ultrafast processes with graphene is not fully understood, yet. We estimate the effect as best we can, a significant degree of uncertainty remains. We use Ref. [8] as a starting point. They report an increase in temperature from room temperature to 1500 K (increase of 1200 K, with plenty of uncertainty) if inter-band transitions are possible. The electron gas temperature is determined by the energy dissipated over one electron-lattice relaxation constant, approximately 1 ps. Since the experiment used femto-second pulses, the entire energy absorbed from one pulse contributed to the electron gas heating. Assuming an absorption of 2.3% (intra-band absorption of a graphene monolayer [8]) from a pulse fluence of $700\,\mathrm{mJ/m^2}$ corresponds to an absorbed energy density of $15\,\mathrm{mJ/m^2}$ in the reference in the inter-band regime. In contrast, we have pulses of at least 10 ps in mind, so we must compare to the product of the relaxation time and the *power* absorbed per unit area of graphene in the waveguide.

In order to get an estimate for our proposal, we assume an optical decay length of 1 mm, which is a realistic number in the low-loss regime $E_F \geq 0.4\,\mathrm{eV}$ at room temperature. Together with a strip width of 150 nm, we find an effective dissipated energy per electron-lattice relaxation time of $\approx 5\,\mathrm{mJ/m^2}$ for each Watt of pump power. The resulting electron heating must be based on the reported 1200 K increase. As a result, we would estimate an electron gas heating in the ballpark of 300 K above the lattice temperature per Watt of pump power. This heating effect should drop by an order of magnitude at 60 K due to reduced optical absorption.

In the manuscript, we project pump powers between 10 mW and 10 W. Clearly, the upper boundary becomes problematic due to this effect, but it appears that for pulse powers below 300 mW the impact is minor both at room temperature (leading to 100 K of heating) and at 60 K (leading to 10 K heating). Towards the lower end of projected pump power range, we do not anticipate any significant effect.

In order to demonstrate the impact of thermal imbalance, we repeated the calculation with 60 K lattice temperature and 160 K electron gas temperature, which corresponds to 3 W pump power at that temperature and in the low optical loss regime. We find that the abso-

lute gain drops by an order of magnitude and the relative gain by two orders of magnitude, but remains above the detection threshold. It should be noted that silicon also exhibits negative effects with higher powers. For cw excitation the pump power is limited to the order of 10 mW due to the accumulation of free carriers generated by two-photon absorption. This can be remedied by using sub-ns pulses, but it will be very difficult to go beyond peak powers in the Watt range.

Supplementary References

[1] C. Wolff, M. J. Steel, B. J. Eggleton, and C. G. Poulton, "Stimulated brillouin scattering in integrated photonic waveguides: Forces, scattering mechanisms, and coupled-mode analysis," Phys. Rev. A **92**, 013836 (2015).
[2] C. Wolff, C. Tserkezis, and N. A. Mortensen, "Enhanced ponderomotive force in graphene due to interband resonance," New J. Phys. **21**, 073046 (2019).
[3] T. Stauber, N. M. R. Peres, and A. K. Geim, "Optical conductivity of graphene in the visible region of the spectrum," Phys. Rev. B **78**, 085432 (2008).
[4] Y.-C. Chang, C.-H. Liu, C.-H. Liu, S. Zhang, S. R. Marder, E. E. Narimanov, Z. Zhong, and T. B. Norris, "Realization of mid-infrared graphene hyperbolic metamaterials," Nat. Commun. **7**, 10568 (2016).
[5] R. W. Boyd, *Nonlinear optics, 3rd edition* (Academic Press, 2003).
[6] Ravi Pant, Christopher G. Poulton, Duk-Yong Choi, Hannah Mcfarlane, Samuel Hile, Enbang Li, Luc Thevenaz, Barry Luther-Davies, Stephen J. Madden, and Benjamin J. Eggleton, "On-chip stimulated brillouin scattering," Opt. Express **19**, 8285–8290 (2011).
[7] Raphaël Van Laer, Bart Kuyken, Dries Van Thourhout, and Roel Baets, "Interaction between light and highly confined hypersound in a silicon photonic nanowire," Nat. Photon. **9**, 199 (2015).
[8] Giancarlo Soavi, Gang Wang, Habib Rostami, David G. Purdie, Domenico De Fazio, Teng Ma, Birong Luo, Junjia Wang, Anna K. Ott, Duhee Yoon, Sean A. Bourelle, Jakob E. Muench, Ilya Goykhman, Stefano Dal Conte, Michele Celebrano, Andrea Tomadin, Marco Polini, Giulio Cerullo, and Andrea C. Ferrari, "Broadband, electrically tunable third-harmonic generation in graphene," Nat. Nanotechnol. **13**, 583–588 (2018).
